# Supplementary material for: Diversity of Fast-Growth Spore-Forming Microbes and Their Activity as Plant Partners
Source: Microorganisms. 2023 Jan 17;11(2):232. doi: 10.3390/microorganisms11020232 (PMC9961442; doi:10.3390/microorganisms11020232)
Supplement: Supplementary file 1 [file microorganisms-11-00232-s001.zip › FIgures S1, S2, S3, S4.pdf]

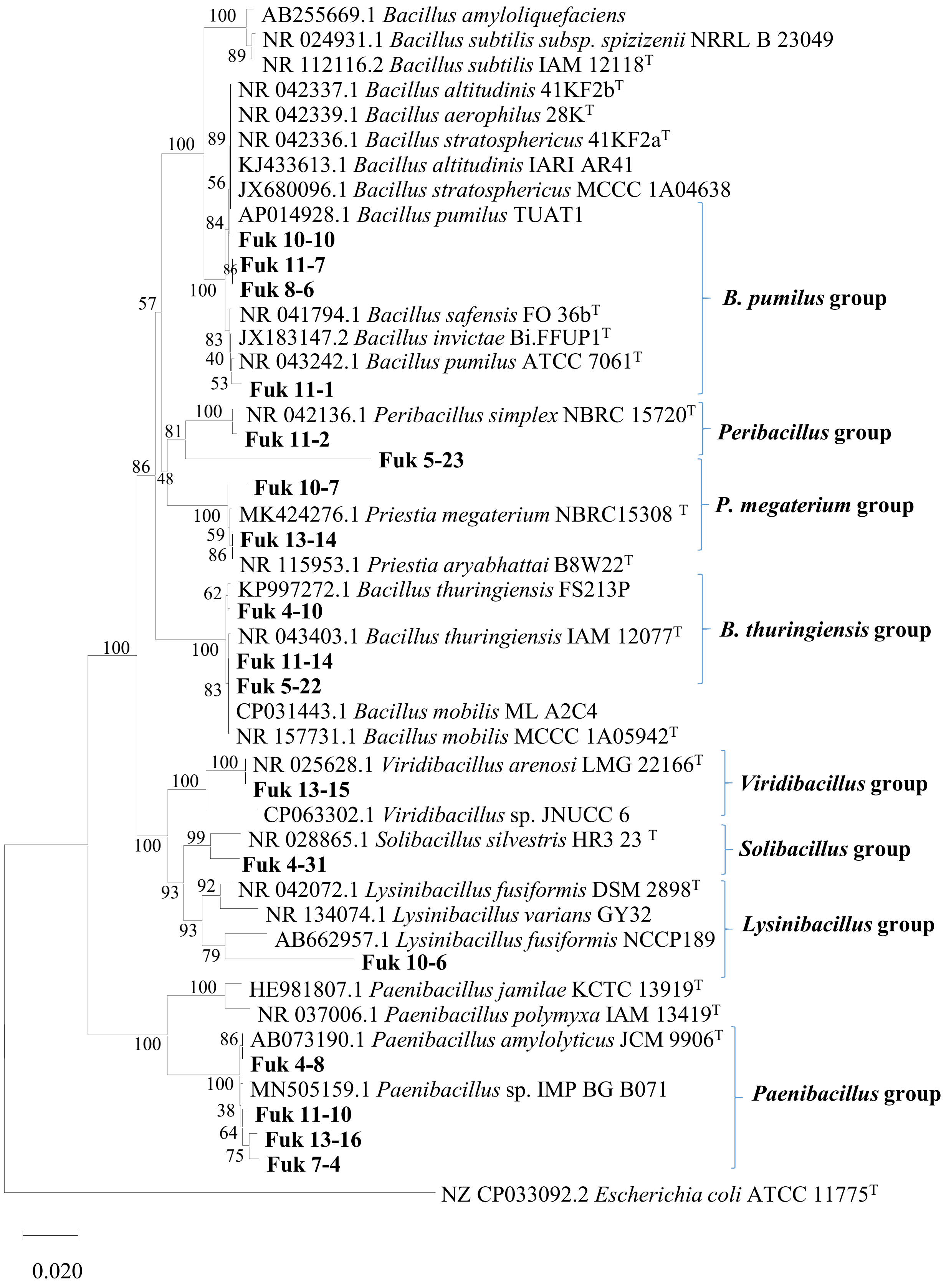

Figure S1. Phylogenetic analysis of Fukushima isolates based on 16S rRNA sequences. The phylogenetic tree included eighteen isolates and reference strains. The tree is based on differences in 1400 bp DNA fragments. The scale bar represents substitutions per nucleotide position, and each genus includes the percentage of the total isolates. Numbers at the nodes indicate the level of bootstrap support (%) based on a neighbor-joining analysis of 1000 re-sampled datasets.

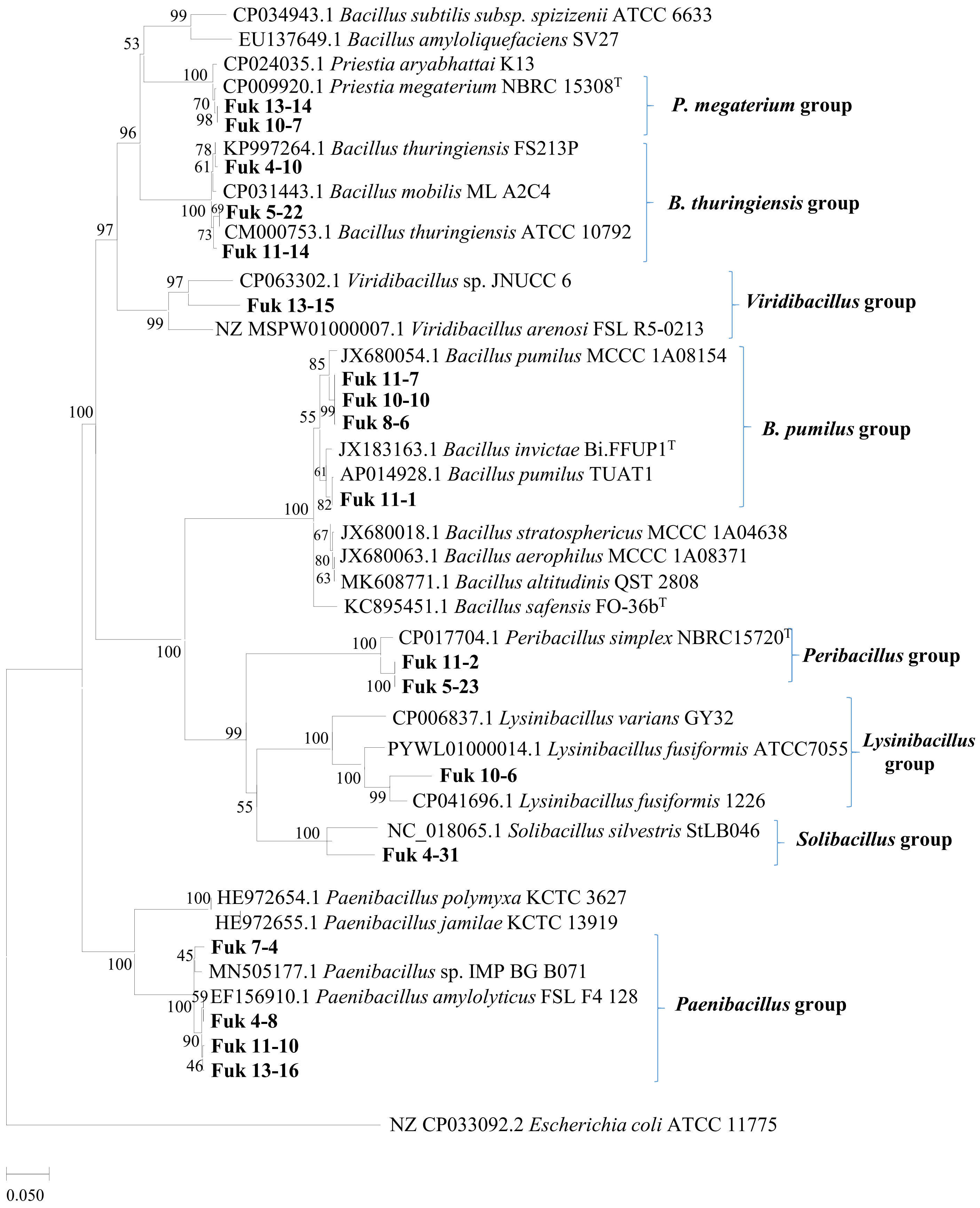

Figure S2. Phylogenetic analysis based on the *rpoB* sequences of Fukushima isolates. The differences are based on a 1300-bp DNA fragment gene. The numbers at the branch nodes indicate bootstrap values (%) based on a neighbor-joining analysis of 1000 re-sampled datasets. The scale bar indicates substitutions per site.

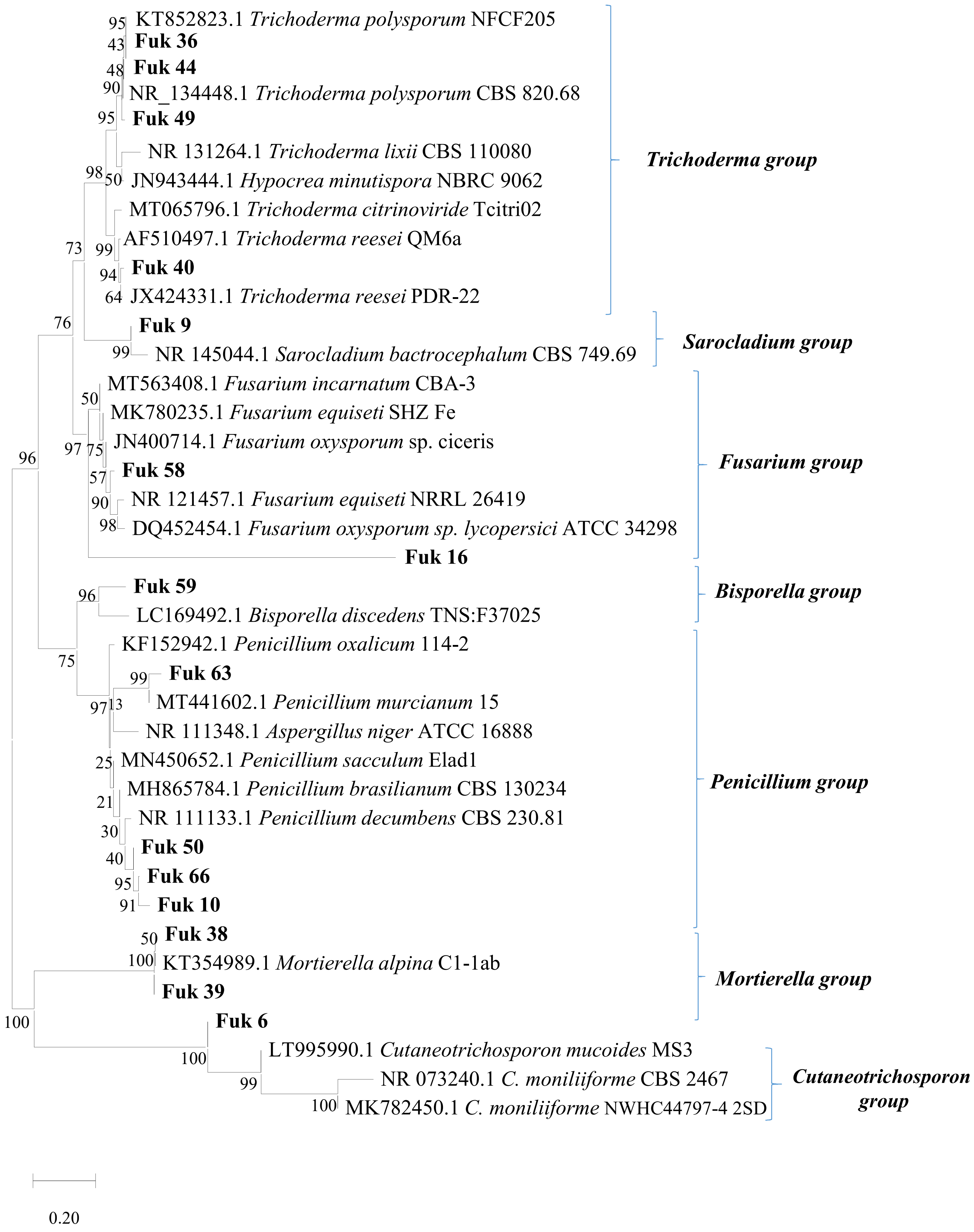

Figure S3. Phylogenetic tree based on ITS sequences of Fukushima isolates, constructed with a 150 bp DNA fragment from sequences. The numbers at the branch nodes indicate bootstrap values (%) based on a neighbor-joining analysis of 1000 re-sampled datasets. The scale bar indicates changes per site.

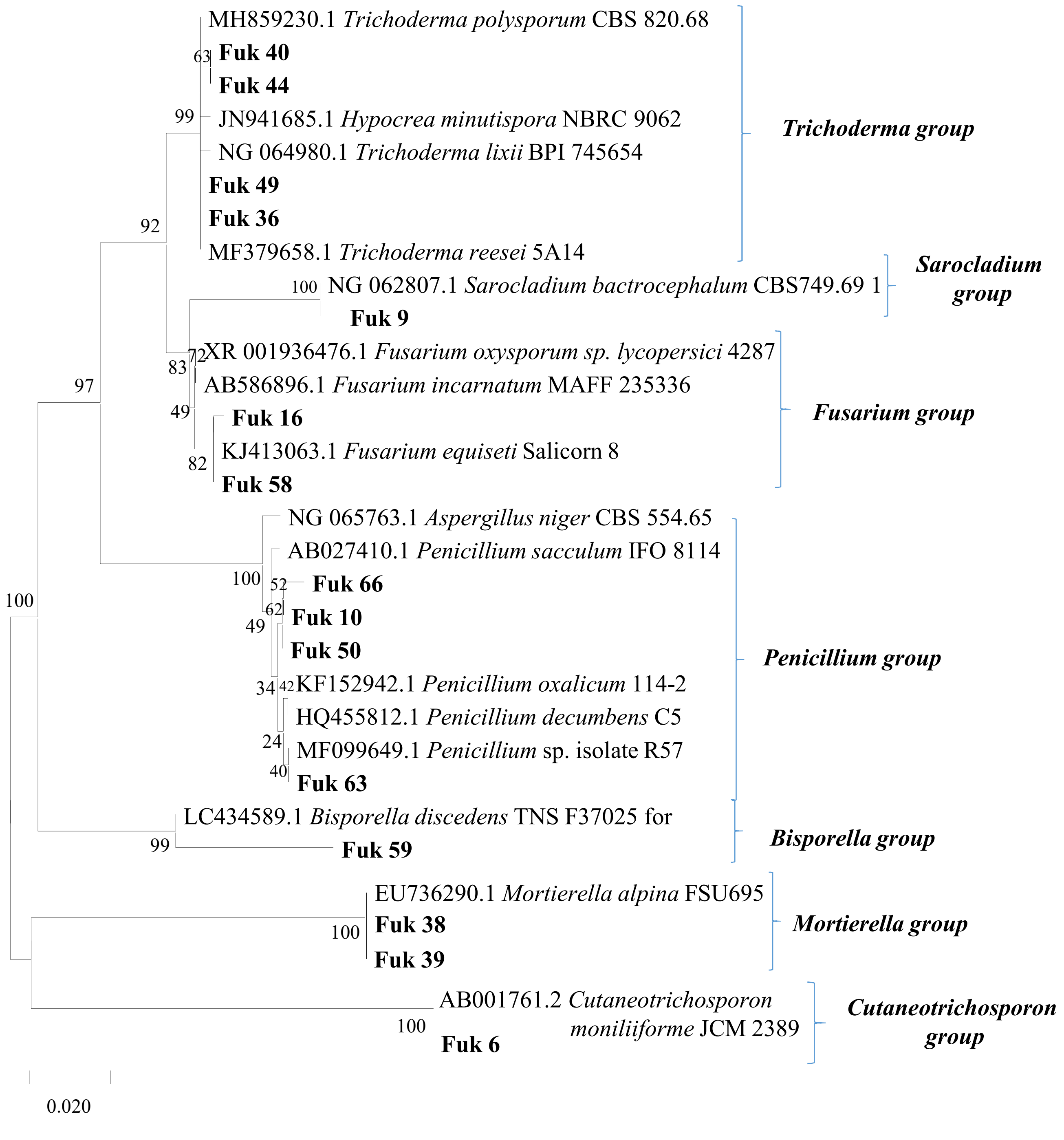

Figure S4. Phylogenetic tree based on 18S rRNA sequences of Fukushima isolates, constructed with 1000 bp DNA fragment gene sequences. The numbers at the branch nodes indicate bootstrap values (%) based on a neighbor-joining analysis of 1000 re-sampled datasets. The scale bar indicates changes per site.
