# Supplementary material for: Diversity of Fast-Growth Spore-Forming Microbes and Their Activity as Plant Partners
Source: Microorganisms. 2023 Jan 17;11(2):232. doi: 10.3390/microorganisms11020232 (PMC9961442; doi:10.3390/microorganisms11020232)
Supplement: Supplementary file 1 [file microorganisms-11-00232-s001.zip › Table S1, Table S2.pdf]

Table S1. Soil and Rhizosphere sample characteristics

Number / Label	Location	Origin	Sample Characteristics	Bacteria	Fungus	Total SFM (cultured)
1	Fukushima	Soil	Forest, Soil under mixed litter	21	4	25
2	Fukushima	Rhizosphere	Field, Japanese pampas grass root	14	3	17
3	Fukushima	Rhizosphere	Litter mix from radioactive area, river area (downstream)	20	4	24
4	Fukushima	Rhizosphere	Forest, Litter mix	37	8	45
5	Fukushima	Soil	Field, Soil under Miscanthus spp.	26	5	31
6	Fukushima	Rhizosphere	River area (downstream), Litter mix	18	4	22
7	Fukushima	Soil	Paddy field, Soil under rice straw	15	3	18
8	Fukushima	Soil	Paddy field, Soil under rice straw and grasses	12	2	14
9	Fukushima	Rhizosphere	Litter mix, river area	14	3	17
10	Fukushima	Rhizosphere	Japanese pampas grass root	10	2	12
11	Fukushima	Soil	Soil under Japanese pampas grass, field	24	5	29
12	Fukushima	Rhizosphere	Forest, Litter mix	3	0	3
13	Fukushima	Rhizosphere	Forest, Litter mix	16	4	20
14	Fukushima	Rhizosphere	Forest, Litter mix	3	0	3
15	Fukushima	Rhizosphere	Field, Miscanthus spp. Litter	20	4	24
16	Fukushima	Rhizosphere	Paddy field, Rice straw	8	2	10
17	Fukushima	Rhizosphere	Field, Japanese pampas grass litter	1	0	1
18	Fukushima	Rhizosphere	Field, Japanese pampas grass root	18	4	22
19	Fukushima	Rhizosphere	Paddy field, Rice straw	17	4	21
20	Fukushima	Rhizosphere	Forest, Litter mix	23	5	28
			**Total**	**320**	**66**	**386**

Table S2. Summary of SFM results under limited nutrients

Group	Sample name	Fe	K	P (inorganic)	N-free	Acid pH (4.5 - 5.5)	Alkaline pH (8 -10)	NaCl (4%)
Fungus	Fuk 6	-	-	1.0	++++	++++	++++	++++
	Fuk 9	-	-	+	++++	++++	++++	++++
	Fuk 10	2.0	-	+	++++	++++	++++	++++
	Fuk 16	-	-	+	++++	++++	++++	++++
	Fuk 36	-	-	+	-	++++	++++	-
	Fuk 38	-	-	+	++++	++++	++++	++++
	Fuk 39	-	-	+	++++	++++	++++	++++
	Fuk 40	-	-	+	-	++++	++++	++++
	Fuk 44	-	-	+	++++	++++	++++	-
	Fuk 49	-	-	-	++++	++++	++++	-
	Fuk 50	-	-	1.1	++++	++++	++++	++++
	Fuk 58	2.0	-	+	++++	++++	++++	++++
	Fuk 59	-	-	1.3	++++	++++	++++	++++
	Fuk 63	-	-	+	-	++++	++++	++++
	Fuk 66	2.0	-	+	++++	++++	++++	++++
Bacteria	Fuk 4_8	-	+	1.0	++++	++++	++++	++++
	Fuk 4_10	-	+	+	++++	++++	++++	++++
	Fuk 4_31	-	+	1.0	++++	++++	++++	++++
	Fuk 5_22	-	+	+	++++	++++	++++	++++
	Fuk 5_23	-	+	1.1	++++	++++	++++	++++
	Fuk 7_4	-	+	1.3	++++	++++	++++	++++
	Fuk 8_6	-	+	1.3	++++	++++	++++	++++
	Fuk 10_6	-	+	1.0	++++	++++	++++	++++
	Fuk 10_7	-	+	+	++++	++++	++++	++++
	Fuk 10_10	-	+	1.0	++++	++++	++++	++++
	Fuk 11_1	-	+	+	++++	++++	++++	++++
	Fuk 11_2	-	+	1.4	++++	++++	++++	++++
	Fuk 11_7	-	-	-	-	-	-	-
	Fuk 11_10	-	+	1.2	++++	-	++++	++++
	Fuk 11_14	-	+	+	++++	++++	++++	++++
	Fuk 13_14	-	+	1.1	++++	++++	++++	++++
	Fuk 13_15	-	-	-	++++	++++	++++	+
	Fuk 13_16	-	+	+	++++	++++	++++	++++

Average response with 3 replication of each test.
- No growth, + could grow, ++++ growth of the strains same or better than control
