# Supplementary material for: Diversity of Fast-Growth Spore-Forming Microbes and Their Activity as Plant Partners
Source: Microorganisms. 2023 Jan 17;11(2):232. doi: 10.3390/microorganisms11020232 (PMC9961442; doi:10.3390/microorganisms11020232)
Supplement: Supplementary file 1 [file microorganisms-11-00232-s001.zip › Table S3. SFM_Bacteria BIOLOG.pdf]

Table S3. Metabolic activity by redox chemistry of Fukushima SF-bacteria

Test / Sample (Group)	*Priestia*		*Bacillus pumilus*				*Bacillus thuringiensis*			*Peribacillus*		*Viridibacillus*	*Lysinibacillus*	*Solibacillus*	*Paenibacillus*			
	FUK10_7	FUK13_14	FUK8_6	FUK10_10	FUK11_1	FUK11_7	FUK4_10	FUK5_22	FUK11_14	FUK5_23	FUK11_2	FUK13_15	FUK10_6	FUK4_31	FUK4_8	FUK7_4	FUK11_10	FUK13_16
H_2O (Control)	-	-	-	-	-	-	-	-	-	-	-	-	-	-	-	-	-	-
α-Cyclodextrin	-	-	+	-	+	+	+	-	+	+	-	-	+	-	-	+	+	-
Dextrin	+	+	+	-	+	-	-	-	+	+	+	+	+	+	-	+	+	-
Glycogen	+	+	+	-	+	+	+	+	+	+	+	+	+	-	+	+	+	+
Tween 40	+	+	+	-	+	-	-	+	+	-	-	+	-	+	+	-	INC	+
Tween 80	-	-	-	-	INC	+	+	-	+	-	-	-	-	+	-	-	-	-
N-Acetyl-D-Galactosamine	+	+	-	-	+	-	-	+	+	-	+	+	INC	-	-	+	+	-
N-Acetyl-D-Glucosamine	+	+	+	-	+	+	+	-	-	-	-	-	-	-	INC	-	+	INC
Adonitol	+	+	+	-	+	+	+	-	-	+	-	-	INC	+	-	-	+	-
L-Arabinose	INC	INC	-	-	-	-	-	-	-	-	-	INC	-	INC	-	-	-	-
D-Arabitol	+	+	+	-	+	+	+	-	+	+	-	+	+	-	+	+	-	+
D-Cellobiose	+	+	+	-	+	+	+	-	-	+	-	+	+	+	-	+	INC	-
i-Erythritol	+	+	+	+	+	+	+	INC	+	+	+	INC	+	+	+	+	INC	+
D-Fructose	+	+	-	-	+	+	+	-	+	+	INC	+	INC	+	-	+	-	-
L-Fucose	+	+	INC	-	+	+	+	+	+	-	-	+	-	+	-	+	-	-
D-Galactose	INC	INC	-	-	-	-	-	+	INC	-	-	INC	-	INC	-	-	-	-
Gentiobiose	+	+	-	-	-	+	+	+	+	+	+	+	+	+	-	+	INC	-
α-D-Glucose	-	-	-	-	+	-	-	-	-	-	-	INC	-	INC	+	INC	-	+
m-Inositol	+	+	-	-	+	-	+	+	-	-	-	+	+	+	-	+	+	-
α-D-Lactose	+	+	+	-	+	+	+	-	-	+	+	+	+	+	-	+	INC	-
Lactulose	+	+	+	-	+	+	+	+	+	+	INC	+	-	+	-	+	INC	-
Maltose	+	+	-	-	+	+	+	-	+	+	INC	-	+	+	+	+	+	+
D-Mannitol	+	+	+	-	-	+	+	-	+	+	-	-	+	+	-	+	-	-
D-Mannose	-	-	INC	-	+	INC	INC	-	INC	-	-	-	-	-	-	-	-	-
D-Melibiose	+	+	-	-	+	+	+	-	+	+	+	+	+	+	-	+	-	-
β-Methyl-D-Glucoside	+	+	INC	+	+	+	+	INC	+	+	+	+	+	+	+	+	+	+
D-Psicose	-	-	-	-	-	-	-	-	-	-	-	-	-	-	-	-	-	-
D-Raffinose	+	+	-	-	+	+	+	+	+	+	+	+	INC	+	-	+	-	-
L-Rhamnose	INC	INC	-	-	+	INC	-	+	+	-	-	INC	-	INC	-	+	-	-
D-Sorbitol	+	+	-	-	+	+	+	+	+	+	+	+	+	-	-	-	+	-
Sucrose	+	+	-	-	+	+	+	-	+	+	-	+	+	-	-	+	+	-
D-Trehalose	+	+	+	-	+	+	+	+	-	+	+	+	+	-	-	+	+	-
Turanose	+	+	+	-	+	+	-	+	+	INC	INC	+	INC	+	-	+	INC	-
Xylitol	+	+	-	-	-	+	+	-	+	+	-	+	+	+	-	+	-	-
Pyruvic acid Methyl Ester	INC	INC	+	-	INC	+	+	-	-	-	-	+	-	+	-	-	-	-
Succinic acid Mono- Methyl Ester	+	+	+	-	-	+	+	-	+	-	-	-	-	+	-	+	-	-
Acetic acid	-	-	+	+	+	+	+	-	+	+	+	-	+	+	+	-	+	+
Cis-Aconitic acid	-	-	+	+	+	+	+	-	+	+	-	+	+	+	INC	+	-	INC
Citric acid	+	+	+	+	+	+	+	-	+	+	+	-	+	+	-	-	+	-
Formic acid	-	-	+	-	+	INC	-	+	+	+	+	-	+	+	+	-	+	+
D-Galactonic acid Lactone	+	+	-	-	+	-	+	+	-	-	-	INC	INC	+	-	-	-	-
D-Galacturonic acid	+	+	+	-	INC	+	+	-	INC	-	-	+	-	-	-	-	-	-
D-Gluconic acid	+	+	-	-	+	+	-	-	-	+	-	+	+	+	-	+	+	-
Glucosaminic acid	+	+	-	-	+	+	+	+	+	+	-	INC	+	+	-	+	+	-
D-Glucuronic acid	+	+	+	-	+	+	+	+	+	-	-	+	-	+	-	-	-	-
γ-Hydroxybutyric acid	-	-	+	-	-	+	+	-	-	+	-	-	+	+	-	-	-	-
β-Hydroxybutyric acid	+	+	+	-	-	+	+	-	-	+	-	-	+	+	INC	INC	-	INC
α-Hydroxybutyric acid	+	+	+	-	+	+	+	-	-	+	-	-	+	+	-	+	INC	-
p-Hydroxyphenlyacetic acid	+	+	+	-	+	+	+	-	+	+	+	+	INC	+	+	+	+	+
Itaconic acid	+	+	+	-	+	-	-	-	+	+	+	-	+	-	INC	-	+	INC
α-Ketobutyric acid	+	+	-	-	+	+	+	+	+	INC	+	+	+	+	+	INC	+	+
α-Ketoglutaric acid	+	+	+	-	+	INC	+	+	+	+	-	INC	+	-	+	+	+	+
α-Ketovaleric acid	+	+	-	-	+	+	+	+	+	-	-	+	+	-	+	+	+	+
D,L-Lactic acid	-	-	+	-	+	INC	+	-	INC	+	-	+	+	-	-	+	-	-
Malonic acid	+	+	+	-	+	INC	+	+	+	+	-	+	+	+	-	+	-	-
Propionic acid	+	+	+	-	+	INC	+	+	-	+	+	-	+	+	+	+	+	+
Quinic acid	+	+	+	-	INC	+	+	-	+	-	-	-	+	+	-	+	-	-
D-Saccharic acid	+	+	+	-	+	+	-	-	-	+	-	+	+	+	-	+	-	-
Sebacic acid	+	+	+	-	+	+	-	-	+	-	-	+	-	+	-	+	-	-
Succinic acid	+	+	+	-	-	+	+	-	-	-	-	-	INC	+	-	-	-	-
Bromosuccinic acid	+	+	+	INC	+	+	+	-	+	+	+	+	+	+	+	+	+	+
Succinamic acid	+	+	+	-	+	+	+	-	+	-	-	+	INC	+	+	+	INC	+
Glucuronamide	INC	INC	INC	-	+	INC	-	-	+	-	-	-	-	-	-	-	-	-
L-Alaninamide	+	+	+	-	+	+	+	+	+	INC	INC	+	+	-	-	+	INC	-
D-Alanine	+	+	+	-	+	+	-	-	+	+	-	-	+	+	-	-	+	-
L-Alanine	+	+	+	-	+	+	+	-	+	+	-	+	+	-	-	+	+	-
L-Alanyl-Glycine	+	+	+	-	+	+	+	-	-	+	-	+	+	-	-	+	+	-
L-Asparagine	+	+	+	-	+	+	+	+	-	+	-	-	+	-	-	+	INC	-
L-Aspartic acid	+	+	+	-	+	+	+	-	+	+	-	INC	+	+	+	+	+	+
L-Glutamic acid	+	+	+	-	INC	+	+	-	-	+	-	INC	+	-	-	+	INC	-
Glycyl-L-Aspartic acid	+	+	+	-	INC	+	+	-	+	+	-	-	+	-	-	-	-	-
Glycyl-L-Glutamic acid	-	-	+	-	+	+	+	-	+	+	-	-	+	-	-	+	-	-
L-Histidine	+	+	+	-	+	+	+	-	+	+	+	+	+	+	-	+	+	-
Hydroxy-L proline	+	+	+	-	+	+	+	-	+	INC	+	+	+	-	-	+	INC	-
L-Leucine	-	-	+	-	+	-	+	-	+	+	+	+	+	+	INC	INC	+	INC
L-Ornithine	-	-	-	-	+	+	+	+	+	+	+	+	+	-	-	-	+	-
L-Phenylalanine	+	+	-	-	+	+	+	+	+	+	-	-	+	-	-	-	-	-
L-Proline	+	+	+	-	+	+	-	-	+	+	-	INC	+	+	-	+	INC	-
L-Pyroglutamic acid	+	+	+	-	+	+	-	-	+	+	INC	+	+	-	-	+	-	-
D-Serine	+	+	+	-	+	+	+	+	-	+	INC	-	+	+	-	-	-	-
L-Serine	+	+	+	-	INC	+	+	-	-	+	-	-	+	+	-	-	-	-
L-Threonine	+	+	+	-	INC	+	+	-	-	+	-	-	+	-	-	INC	INC	-
D,L-Carnitine	+	+	+	-	+	+	+	-	-	+	INC	-	+	+	INC	INC	INC	INC
γ-Aminobutyric acid	+	+	+	-	-	+	+	-	-	+	-	-	INC	+	-	-	-	-
Urocanic acid	+	+	+	-	+	+	+	-	+	-	-	+	+	+	INC	+	-	-
Inosine	+	+	+	-	+	+	+	+	+	+	+	+	+	+	-	+	+	-
Uridine	+	+	+	-	+	+	-	+	+	+	+	-	+	+	-	-	+	-
Thymidine	+	+	+	-	+	+	+	+	+	+	-	-	+	-	-	-	-	-
Phenylethyl-amine	+	+	+	-	-	INC	-	+	INC	-	+	-	-	-	-	-	+	-
Putrescine	+	+	+	-	+	+	+	+	+	+	+	-	+	-	-	+	+	-
2-Aminoethanol	+	+	+	-	+	+	+	+	+	+	+	INC	+	-	-	INC	+	-
2,3-Butanediol	+	+	+	-	+	+	+	-	-	+	INC	INC	+	-	-	+	INC	-
Glycerol	+	+	-	-	+	+	+	+	+	+	+	+	+	+	-	+	+	-
D,L,α-Glycerol-Phosphate	+	+	+	INC	-	+	+	-	-	+	+	-	+	-	-	INC	+	-
α-D-Glucose-1-Phosphate	+	+	+	+	+	+	+	-	-	+	INC	-	+	+	-	+	INC	-
D-Glucose-6-Phosphate	+	+	+	INC	+	+	+	-	INC	+	-	+	+	+	INC	INC	-	INC

INC: inconclusive resutls due to the time response (some took more than week)
+ : positive reaction
-: no reaction.
