# Supplementary material for: Diversity of Fast-Growth Spore-Forming Microbes and Their Activity as Plant Partners
Source: Microorganisms. 2023 Jan 17;11(2):232. doi: 10.3390/microorganisms11020232 (PMC9961442; doi:10.3390/microorganisms11020232)
Supplement: Supplementary file 1 [file microorganisms-11-00232-s001.zip › Table S4. SFM_Fungus BIOLOG.pdf]

Table S4. Metabolic activity by redox chemistry of Fukushima fungi.

Test / Sample (Group)	*Trichoderma*				*Sarocladium*	*Fusarium*		*Bisporella*	*Penicillium*				*Mortierella*		*Cutaneo-trichosporon*
	FUK 36	FUK 49	FUK 40	FUK 44	FUK 9	FUK 58	FUK 16	FUK 59	FUK 50	FUK 63	FUK 66	FUK 10	FUK 39	FUK 38	FUK 6
H_2O (Control)	-	-	-	-	+	INC	INC	INC	-	-	INC	INC	+	+	-
α-Cyclodextrin	+	-	INC	INC	+	+	+	-	-	-	+	+	-	+	+
Dextrin	+	+	+	+	+	+	+	+	+	+	+	+	+	-	+
Glycogen	+	+	+	+	+	+	+	+	+	+	+	+	+	+	+
Tween 40	+	+	+	+	+	+	+	-	+	+	+	+	+	+	+
Tween 80	+	+	+	+	+	+	+	-	+	+	-	+	+	+	+
N-Acetyl-D-Galactosamine	+	+	+	+	-	+	+	-	+	-	INC	+	-	+	INC
N-Acetyl-D-Glucosamine	+	+	+	+	+	+	+	-	+	+	+	+	-	+	+
Adonitol	+	-	INC	INC	+	+	+	+	-	+	+	INC	-	+	INC
L-Arabinose	-	-	-	-	-	-	-	INC	-	-	-	-	-	-	-
D-Arabitol	+	-	INC	INC	-	+	+	-	-	-	INC	+	-	-	+
D-Cellobiose	-	-	-	-	-	+	+	-	-	-	-	-	-	+	+
i-Erythritol	+	-	INC	INC	+	+	+	+	+	+	+	+	-	+	+
D-Fructose	+	+	+	+	-	+	+	+	+	-	+	-	+	+	+
L-Fucose	-	-	-	-	-	+	+	+	-	+	-	-	+	+	INC
D-Galactose	-	-	-	-	-	+	-	-	-	-	-	-	+	-	-
Gentiobiose	+	+	+	+	-	+	+	-	-	+	INC	+	-	-	+
α-D-Glucose	-	+	INC	INC	-	-	-	INC	-	-	-	-	-	-	-
m-Inositol	+	-	INC	INC	+	+	+	-	-	-	-	+	-	-	+
α-D-Lactose	+	-	INC	INC	INC	+	+	+	+	+	+	+	-	+	+
Lactulose	+	+	+	+	INC	+	+	-	-	-	-	+	-	+	+
Maltose	+	+	+	+	-	+	+	-	-	INC	-	+	+	-	+
D-Mannitol	INC	-	INC	INC	-	-	+	-	-	INC	-	+	-	-	+
D-Mannose	INC	-	INC	INC	-	+	-	-	-	+	INC	-	-	-	-
D-Melibiose	+	+	+	+	+	+	+	+	+	+	+	+	+	+	+
β-Methyl-D-Glucoside	+	+	+	+	-	+	+	-	+	+	-	+	-	+	+
D-Psicose	-	INC	-	INC	-	-	-	-	-	-	INC	-	-	-	-
D-Raffinose	+	-	INC	INC	-	+	+	-	+	+	+	+	-	-	+
L-Rhamnose	+	-	INC	INC	-	+	+	-	-	-	-	-	-	-	INC
D-Sorbitol	+	+	+	+	+	+	+	-	INC	+	-	+	+	-	+
Sucrose	+	+	+	+	-	+	+	-	+	-	-	+	+	+	+
D-Trehalose	+	+	+	+	+	+	+	-	-	-	-	+	+	+	+
Turanose	+	+	+	+	+	+	+	-	-	INC	-	+	+	+	+
Xylitol	+	+	+	+	+	+	+	-	-	-	-	+	-	-	+
Pyruvic acid Methyl Ester	+	-	INC	INC	+	+	+	+	-	INC	INC	+	-	+	INC
Succinic acid Mono- Methyl Ester	+	-	INC	INC	+	+	+	-	-	INC	+	INC	-	+	+
Acetic acid	+	+	+	+	+	+	+	-	+	+	+	+	+	+	+
Cis-Aconitic acid	+	+	+	+	+	+	+	+	+	+	+	+	+	+	+
Citric acid	+	+	+	+	+	+	+	+	-	-	+	+	-	+	+
Formic acid	+	+	+	+	+	+	+	-	+	+	+	+	+	+	+
D-Galactonic acid Lactone	INC	-	INC	INC	+	+	+	+	+	+	+	+	-	+	+
D-Galacturonic acid	INC	-	INC	INC	+	+	+	-	-	+	INC	INC	+	+	INC
D-Gluconic acid	+	+	+	+	+	+	+	-	+	+	+	+	-	+	+
Glucosaminic acid	+	+	+	+	+	+	+	+	+	+	-	+	-	+	+
D-Glucuronic acid	INC	INC	INC	INC	+	-	-	-	-	+	-	INC	-	+	INC
γ-Hydroxybutyric acid	+	+	INC	+	+	+	+	-	+	-	-	+	-	+	+
β-Hydroxybutyric acid	INC	-	INC	INC	+	+	+	-	+	+	-	+	-	+	+
α-Hydroxybutyric acid	+	+	+	+	+	+	+	+	+	+	+	+	-	+	+
p-Hydroxyphenlyacetic acid	+	-	INC	INC	+	+	+	-	+	+	+	+	+	+	+
Itaconic acid	+	+	+	+	-	+	+	-	+	+	+	+	+	+	+
α-Ketobutyric acid	+	+	+	+	+	+	+	+	+	+	+	+	+	-	+
α-Ketoglutaric acid	+	+	+	+	+	+	+	-	+	+	+	+	+	INC	+
α-Ketovaleric acid	+	+	+	+	+	+	+	-	-	+	-	+	+	+	+
D,L-Lactic acid	+	-	+	+	+	+	+	-	+	+	+	+	-	+	+
Malonic acid	+	+	+	+	+	+	+	-	+	+	+	+	+	+	+
Propionic acid	+	+	+	+	+	+	+	-	+	+	+	+	+	+	+
Quinic acid	+	+	+	+	+	+	+	-	+	+	+	+	-	-	+
D-Saccharic acid	+	+	+	+	+	+	+	-	-	+	-	INC	-	-	+
Sebacic acid	-	-	-	INC	+	-	+	-	+	+	-	INC	-	-	+
Succinic acid	INC	-	INC	INC	+	+	+	-	-	+	INC	INC	-	-	-
Bromosuccinic acid	+	+	+	+	+	+	+	-	+	INC	+	-	+	+	+
Succinamic acid	+	-	INC	INC	INC	+	+	+	+	+	+	+	+	+	+
Glucuronamide	-	+	-	INC	-	-	-	+	-	-	-	-	-	INC	-
L-Alaninamide	+	+	+	+	+	+	+	-	+	-	-	+	+	+	+
D-Alanine	+	+	+	+	+	+	+	-	+	+	+	+	-	-	+
L-Alanine	+	+	+	+	+	+	+	-	+	+	+	+	+	-	+
L-Alanyl-Glycine	+	+	+	+	+	INC	INC	-	+	+	+	+	+	+	+
L-Asparagine	+	+	+	+	-	INC	INC	-	+	+	+	+	-	-	+
L-Aspartic acid	+	+	+	+	INC	INC	INC	-	-	+	+	+	-	-	+
L-Glutamic acid	+	+	+	+	+	+	+	-	+	+	+	+	-	-	+
Glycyl-L-Aspartic acid	+	+	+	+	+	+	+	-	-	+	INC	+	-	-	+
Glycyl-L-Glutamic acid	+	+	+	+	+	+	+	-	INC	INC	+	-	+	INC	+
L-Histidine	+	+	+	+	+	+	+	-	-	+	-	+	+	+	+
Hydroxy-L proline	+	+	+	+	-	+	+	-	+	+	+	+	+	+	+
L-Leucine	+	-	INC	INC	+	+	+	+	+	+	+	+	-	+	+
L-Ornithine	+	+	+	+	+	+	+	+	+	+	INC	+	+	+	+
L-Phenylalanine	+	+	+	+	+	+	+	-	INC	+	-	+	+	+	+
L-Proline	+	+	+	+	+	+	+	-	+	INC	-	+	+	+	+
L-Pyroglutamic acid	+	+	+	+	-	+	+	-	INC	-	+	+	+	-	+
D-Serine	+	+	+	+	-	+	+	-	INC	-	+	+	+	INC	+
L-Serine	+	+	+	+	+	+	+	-	+	-	INC	+	+	-	+
L-Threonine	+	+	+	+	+	+	+	-	-	+	INC	+	-	-	+
D,L-Carnitine	+	+	+	+	+	+	+	-	-	INC	+	INC	-	-	+
γ-Aminobutyric acid	INC	-	INC	INC	+	-	+	-	-	+	-	+	-	-	+
Urocanic acid	INC	-	INC	INC	+	+	+	+	-	-	+	-	+	+	+
Inosine	+	-	INC	INC	+	+	+	-	+	+	+	+	+	+	+
Uridine	INC	-	INC	INC	+	+	+	-	+	-	-	+	-	+	+
Thymidine	+	+	+	+	+	+	+	-	+	-	INC	+	+	-	+
Phenylethyl-amine	+	+	+	+	+	+	+	-	+	+	+	+	+	INC	+
Putrescine	+	+	+	+	INC	+	+	-	-	+	-	+	-	+	+
2-Aminoethanol	+	+	+	+	+	+	+	-	+	-	INC	+	-	+	+
2,3-Butanediol	+	-	INC	INC	+	+	+	-	+	+	+	+	-	+	+
Glycerol	INC	-	INC	INC	+	+	+	-	+	+	+	+	-	+	+
D,L,α-Glycerol-Phosphate	INC	-	INC	INC	+	+	+	-	+	+	+	+	+	+	+
α-D-Glucose-1-Phosphate	+	-	INC	INC	+	+	+	-	+	+	-	-	-	-	+
D-Glucose-6-Phosphate	INC	-	INC	INC	+	+	+	-	-	+	INC	INC	-	-	INC

INC: inconclusive resutls due to the time to response (some took more than week)
+ : positive reaction
-: no reaction.
